# Anticancer Effects of Cepharanthine: an Updated Minireview

**DOI:** 10.7150/jca.118647

**Published:** 2026-04-08

**Authors:** Pei-Yin Yang, Jyun-Xue Wu, Ya-Hui Chen, Shun-Fa Yang, Yi-Tzu Chu, Yi-Hsuan Hsiao

**Affiliations:** 1 Institute of Medicine, Chung Shan Medical University, Taichung, Taiwan.; 2 Department of Obstetrics and Gynecology, Changhua Christian Hospital, Changhua, Taiwan.; 3 Women's Health Research Laboratory, Changhua Christian Hospital, Changhua, Taiwan.; 4 Department of Medical Research, Chung Shan Medical University Hospital, Taichung, Taiwan.; 5 Department of Post-Baccalaureate Medicine, College of Medicine, National Chung-Hsing University, Taichung, Taiwan; 6 School of Medicine, Chung Shan Medical University, Taichung, Taiwan.

**Keywords:** cepharanthine, anticancer activities, programmed cell death, cell cycle arrest, metastasis, angiogenesis, oxidative stress

## Abstract

Cepharanthine (CEP), a bisbenzylisoquinoline alkaloid, has a long history of clinical use in Japan for various diseases. Over the past few decades, it is potent anticancer activities have garnered significant attention. In this review shows CEP exhibits anti-cancer activity via diverse mechanisms: it induces programmed cell death via intrinsic mitochondrial pathways, extrinsic death receptor signaling, and endoplasmic reticulum stress. It also arrests uncontrolled cell proliferation by causing G0/G1 and G2/M phase cell cycle arrest. Furthermore, CEP inhibits tumor progression by suppressing metastasis, invasion, and angiogenesis. It effectively modulates crucial oncogenic signaling pathways such as PI3K/Akt/mTOR, NF-κB, and JAK2/STAT3, MAPK, and plays a role in regulating oxidative stress within cancer cell. Notably, CEP can reverse multidrug resistance by inhibiting drug efflux transporters, thereby enhancing the efficacy of conventional chemotherapies, this review shows the diverse molecular targets of CEP and elucidates the complex network of pathways it modulates, positioning it as a promising natural compound for further investigation in cancer therapy.

## 1. Introduction

Cancer remains one of the most formidable public health challenges globally, characterized by uncontrolled cell proliferation, invasion, and metastasis. It is a leading cause of mortality worldwide, responsible for nearly one in six deaths (16.8%) and almost one in four deaths (22.8%) from noncommunicable diseases (NCDs) 1. The disease is significant impact on life expectancy imposes considerable societal and economic burdens, which vary depending on cancer type, geographic region, and gender 2. For instance, a study in 2020 estimated that one million children lost their mothers to cancer, with breast and cervical cancers accounting for nearly half of these maternal deaths 3. The complexity and heterogeneity of cancer necessitate a continuous search for novel therapeutic strategies, including those derived from natural sources. Natural products have historically been a rich reservoir of bioactive compounds for drug discovery, with many approved anticancer drugs originating from plants, microorganisms, or marine organisms 4. These compounds often exhibit unique chemical structures and diverse mechanisms of action, targeting multiple pathways involved in cancer progression with potentially fewer side effects compared to synthetic drugs.

**Figure 1:**
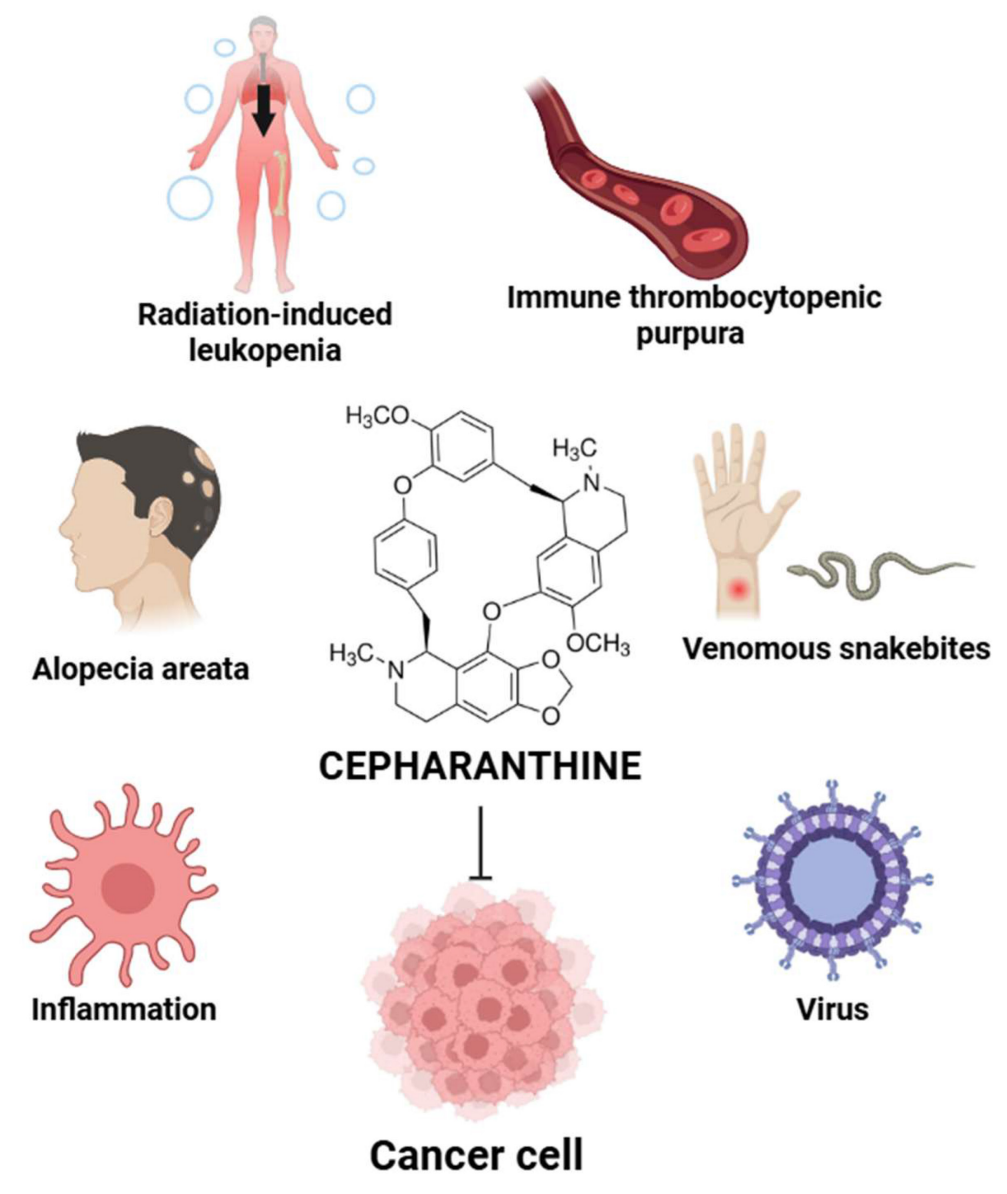
** Overview of CEP therapeutic applications.** CEP has been clinically used for treating radiation-induced leukopenia, immune thrombocytopenic purpura, alopecia areata, and snakebites. It also exhibits anti-inflammatory, antiviral, and anticancer properties. The illustration highlights CEP's chemical structure and its diverse pharmacological effects across multiple disease contexts. (Created with BioRender.com).

Cepharanthine (CEP) (Figure 1) is a bisbenzylisoquinoline alkaloid extracted from the roots of *Stephania cepharantha Hayata* and other *Stephania* species. Traditionally, CEP has been used in Japan for several decades to treat a variety of conditions, such as radiation-induced leukopenia, immune thrombocytopenic purpura 5, alopecia areata 6, 7, and venomous snakebites 8. Beyond these applications, extensive research has illuminated a broad spectrum of pharmacological properties for CEP, including potent anti-inflammatory 9, antiviral 6, 10, and, most notably, antitumor activities 11-15.

The pharmacokinetics of cepharanthine (CEP), a biscoclaurine alkaloid, vary significantly depending on the route of administration. While it has been used clinically in Japan for decades, its low oral bioavailability remains a primary challenge for its therapeutic application 16-18. Cepharanthine (CEP) is characterized by an excellent safety profile and has been used clinically in Japan for over 70 years to treat various acute and chronic conditions without major side effects 19. The sources consistently describe it as a well-tolerated drug where undesirable effects are insignificant and very rarely reported 7.

This review aims to provide a comprehensive overview of the anticancer mechanisms of CEP, focusing on it is molecular targets and the signaling pathways it modulates across various cancer types, drawing from existing literature and incorporating recent findings.

## 2. Anticancer Action of Cepharanthine

**Figure 2:**
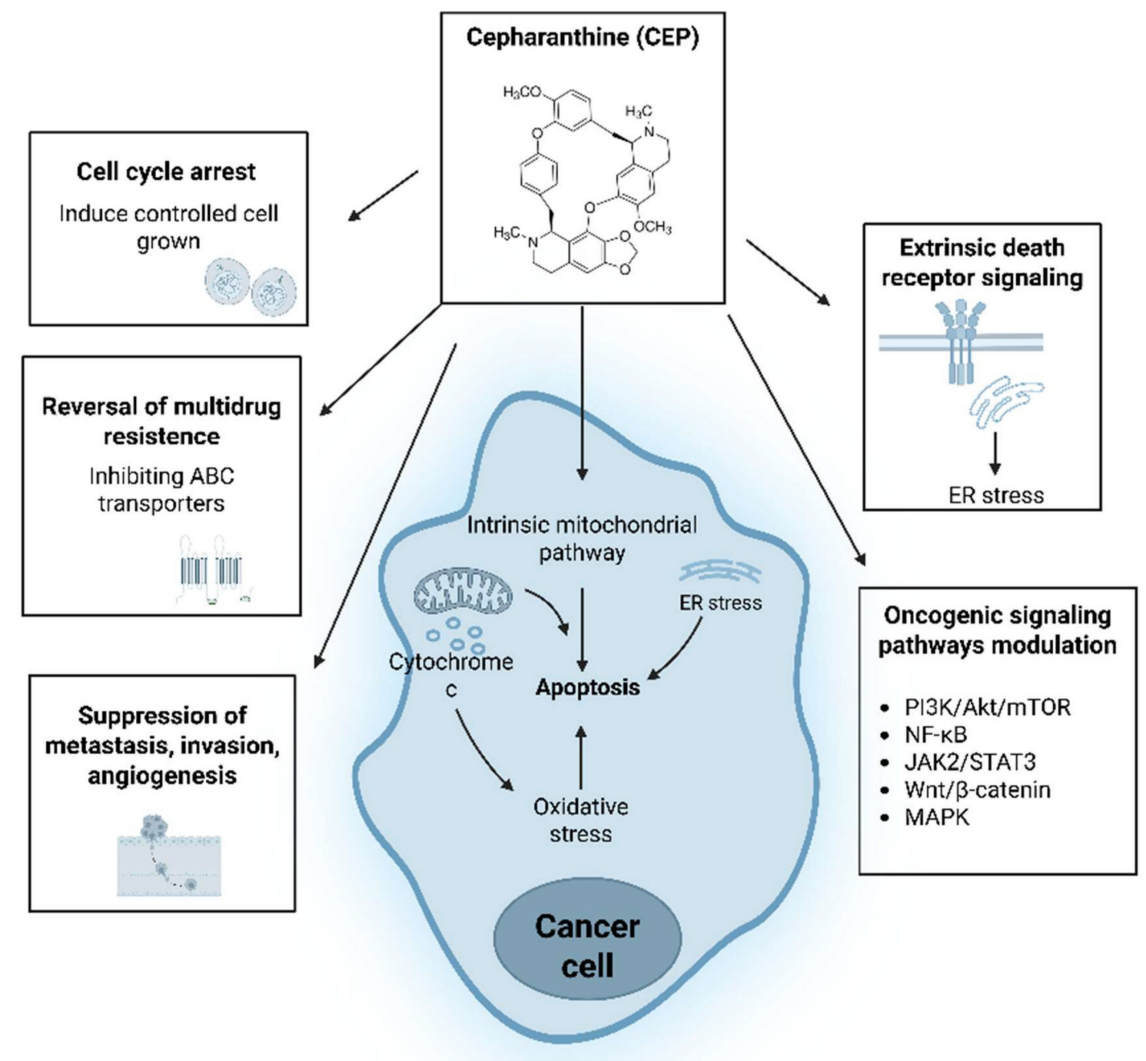
** Schematic overview of Cepharanthine (CEP) anticancer mechanisms.** CEP exerts multifaceted antitumor effects: it induces intrinsic mitochondrial apoptosis through reactive oxygen species (ROS) generation, cytochrome c release, endoplasmic reticulum (ER) stress, and extrinsic death-receptor signaling. On the plasma membrane, CEP reverses multidrug resistance by inhibiting ABC transporters ABCC10/MRP7 and ABCB1/MDR1, thereby increasing intracellular drug accumulation. Additional actions include induction of cell cycle arrest, suppression of metastasis, invasion, and angiogenesis, and modulation of key oncogenic pathways—PI3K/Akt/mTOR, NF-κB, JAK2/STAT3, Wnt/β-catenin, and MAPK—to collectively inhibit cancer cell proliferation and survival. (Created with BioRender.com).

CEP exerts its anticancer effects through a complex interplay of mechanisms (Figure 2), targeting various hallmarks of cancer. These include the induction of programmed cell death, inhibition of cell proliferation via cell cycle arrest, suppression of tumor invasion and metastasis, anti-angiogenic effects, and modulation of cellular stress responses. Furthermore, CEP has been shown to sensitize cancer cells to conventional therapies, offering a potential avenue to overcome drug resistance.

### 2.1 Intrinsic Pathway Activation

The intrinsic pathway of apoptosis is often initiated by intracellular stress signals, leading to mitochondrial outer membrane permeabilization (MOMP). This results in the release of cytochrome c and other pro-apoptotic factors from the mitochondria into the cytoplasm, subsequently activating caspase-9 and caspases-3 and -7, which execute apoptosis. In hepatocellular carcinoma (HCC) cells (Huh7, HepG2), CEP treatment led to mitochondrial dysfunction, characterized by loss of mitochondrial membrane potential (ΔΨ m), and subsequent ROS generation, causing apoptosis 11, 20. specifically showed that CEP triggers apoptosis in HuH-7 cells via activation of JNK1/2 and downregulation of Akt, upstream events that can contribute to mitochondrial-mediated apoptosis 20. For colorectal cancer (CRC) cells (HT-29, SW620, HCT116, COLO-205), CEP induced apoptosis, which was associated with the downregulation of the anti-apoptotic protein Bcl-2 21, Bcl-2 family proteins are critical regulators of MOMP, with anti-apoptotic members (e.g., Bcl-2, Bcl-xL) preventing, and pro-apoptotic members (e.g., Bax, Bak) promoting, cytochrome c release, human ovarian cancer cells, in SKOV3, OVCAR8, A2780 cells induced apoptosis through the mitochondrial pathway, evidenced by increased Bax/Bcl-2 ratio, cytochrome c release, and caspase-3/7 activation 12. Recent studies, on triple-negative breast cancer (TNBC) cells showed that CEP can induce cofilin oxidation-mediated mitochondrial fission and apoptosis, thereby sensitizing these aggressive cells to epirubicin 22, mitochondrial fission frequently occurs prior to MOMP, as well as CEP downregulates TOM20 and TOM70 in colorectal cancer, leading to mitochondrial damage and suppression of the NRF2 signaling pathway. This promotes ROS accumulation and induces ferroptosis, significantly reducing CRC cell viability. Restoring TOM70 reverses these effects. Then Cep triggers ferroptosis through TOM inhibition and NRF2 inactivation, the TOM protein family plays a key role in regulating mitochondrial homeostasis and is frequently overexpressed in various tumor tissues. Its expression is associated with patient prognosis, suggesting that TOM proteins may serve as potential therapeutic targets for modulating mitochondrial function^23^.

### 2.2 Extrinsic Pathway Involvement

The extrinsic pathway is initiated by the binding of death ligands (e.g., FasL, TNF-α) to their cognate death receptors on the cell surface, leading to the activation of caspase-8. While less commonly reported for CEP compared to the intrinsic pathway, some studies suggest its potential involvement. For instance, in renal carcinoma cells, CEP has been shown to significantly enhance TRAIL (Tumor necrosis factor-related apoptosis-inducing ligand)-mediated apoptosis. Although CEP or TRAIL alone had minimal effect, their combination markedly increased apoptotic cell death. This sensitization effect involves CEP promoting the downregulation of anti-apoptotic proteins like survivin and cellular FLICE-inhibitory protein 24, thereby lowering the threshold for extrinsic pathway activation by TRAIL. This demonstrates a clear modulatory role for CEP within the extrinsic apoptotic cascade in this specific cancer context.

### 2.3 Endoplasmic Reticulum (ER) Stress-Induced Apoptosis

ER stress occurs when misfolded proteins accumulate in the ER lumen, triggering the unfolded protein response. Prolonged or severe ER stress can lead to apoptosis. Some natural compounds induce apoptosis via this pathway. While direct, extensive evidence for CEP-induced ER stress leading to apoptosis as a primary mechanism, its impact on calcium homeostasis and ROS generation could indirectly trigger ER stress. For example, in glioblastoma cells, CEP-induced ROS stress 25 could contribute to ER stress, in lung cancer cells, CEP has been shown to trigger robust ER stress by inhibiting NRF2, which also initiates ferroptosis. This CEP-induced ER stress is characterized by the upregulation of ER stress markers like GRP78 and CHOP 26, and it significantly contributes to the apoptotic demise of lung cancer cells and a reduction in their cancer stem cell characteristics. This demonstrates a more direct role of CEP in leveraging ER stress to induce cancer cell apoptosis 27.

## 3. Cell Cycle Arrest

**Figure 3:**
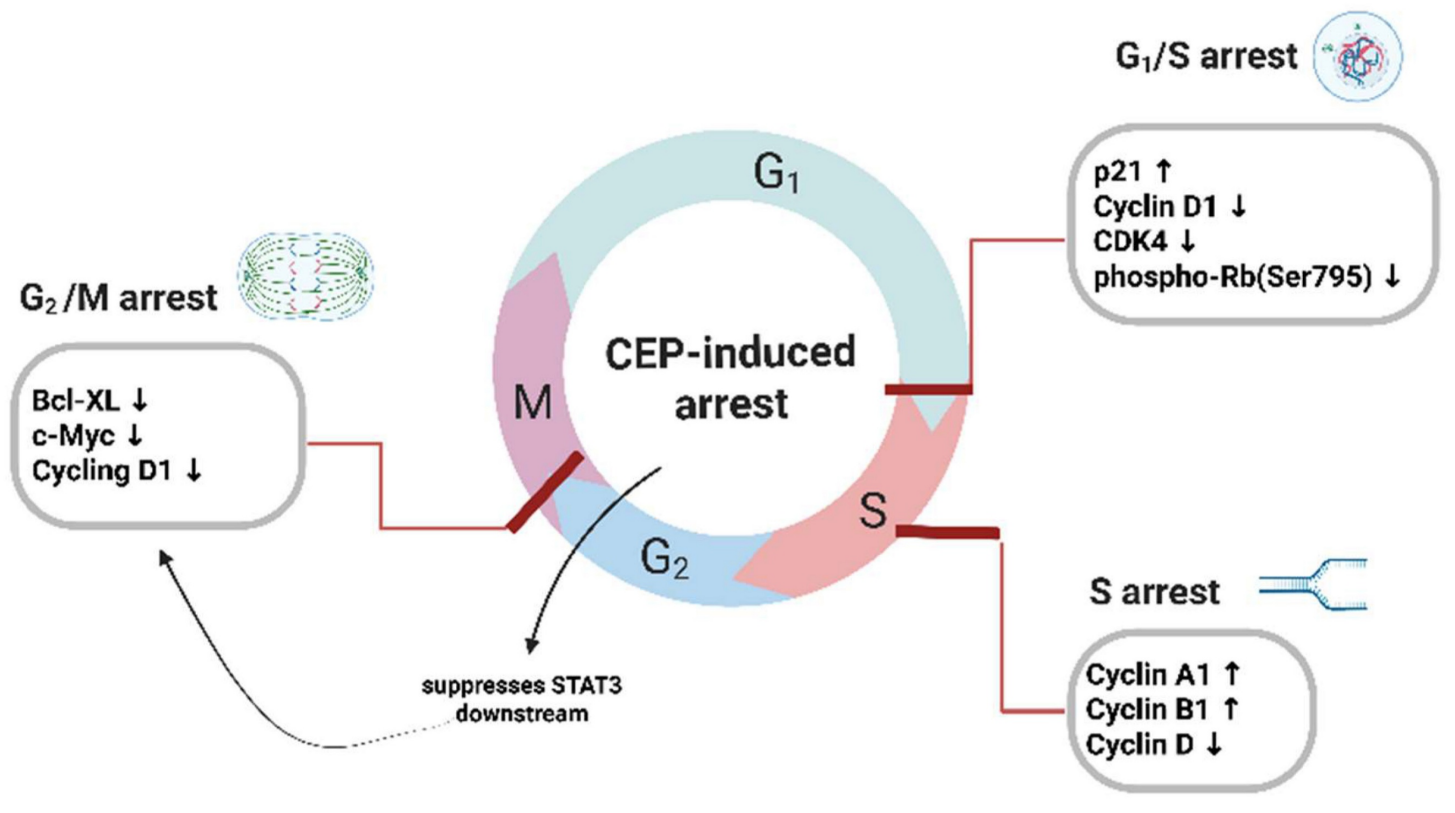
** CEP-mediated blockade of cell cycle progression at multiple checkpoints.** Exposure of cancer cells to Cepharanthine (CEP) results in robust inhibition of cell cycle transitions. At the G₁/S boundary, CEP elevates the CDK inhibitor p21 while concurrently suppressing Cyclin D1, CDK4 and phosphorylation of Rb at Ser795, thereby preventing entry into S phase. Within S phase, CEP further disrupts DNA replication by increasing Cyclin A1 and Cyclin B1 levels alongside a reduction in Cyclin D. Finally, CEP enforces G₂/M arrest through downregulation of STAT3 downstream effectors—namely Bcl-xL, c-Myc and Cyclin D1—effectively blocking progression into mitosis. Together, these coordinated alterations in cyclins, CDKs and anti-apoptotic proteins underlie the multifaceted cell cycle arrest induced by CEP. (Created with BioRender.com).

In addition to inducing apoptosis, cepharanthine can inhibit tumor growth by arresting the cell cycle, uncontrolled cell proliferation is a fundamental characteristic of cancer, driven by dysregulation of the cell cycle. The cell cycle is tightly controlled by cyclins, cyclin-dependent kinases (CDKs), and CDK inhibitors (CKIs). CEP has been shown to induce cell cycle arrest at different checkpoints, thereby inhibiting cancer cell proliferation (Figure 3). G1/S phase arrest: In breast cancer cells, CEP induced G0/G1 cell cycle arrest by upregulating p21 and downregulating Cyclin D1, Cyclin A 12 CDK4, and phospho-Rb (Ser795) 13, and can induce G1/S phase arrest and DNA breakage, inhibit the growth of a variety of cancer cell, including human adenosquamous carcinoma cell line (TYS) 28, human osteosarcoma cell line (SaOS2) 29, CEP induces the expression of cyclin-dependent kinase (CDK) inhibitors, thereby inhibiting the STAT3 signaling pathway that have demonstrated that CEP suppresses STAT3 downstream targets—specifically the anti-apoptotic gene Bcl-xL and the cell-cycle regulators c-Myc and cyclin D1 30, In addition, DNA breaks during cell proliferation by blocking the G1 phase, S phase, and G1 to S phase transition process^12^, ^31^. G2/M Phase Arrest: CEP also exerts its antiproliferative effects by inducing cell cycle arrest at the G2/M phase, usually arresting cells in the G1 and S phases. Jurkat T cells treated with CEP showed a dose-dependent inhibition of cell cycle progression in the S phase, which was accompanied by an upregulation of cyclin A2 and cyclin B1, and a downregulation of cyclin D1 32, These findings underscore the multifaceted role of CEP in modulating cell cycle dynamics across various tumor types. By targeting key regulatory proteins and checkpoints, CEP disrupts the orderly progression of the cell cycle, thereby exerting significant antiproliferative effects. This mechanistic diversity highlights its therapeutic potential not only as a cytotoxic agent but also as a cell cycle modulator capable of acting at multiple regulatory nodes.

## 4. Anti-Metastatic Mechanisms

**Figure 4:**
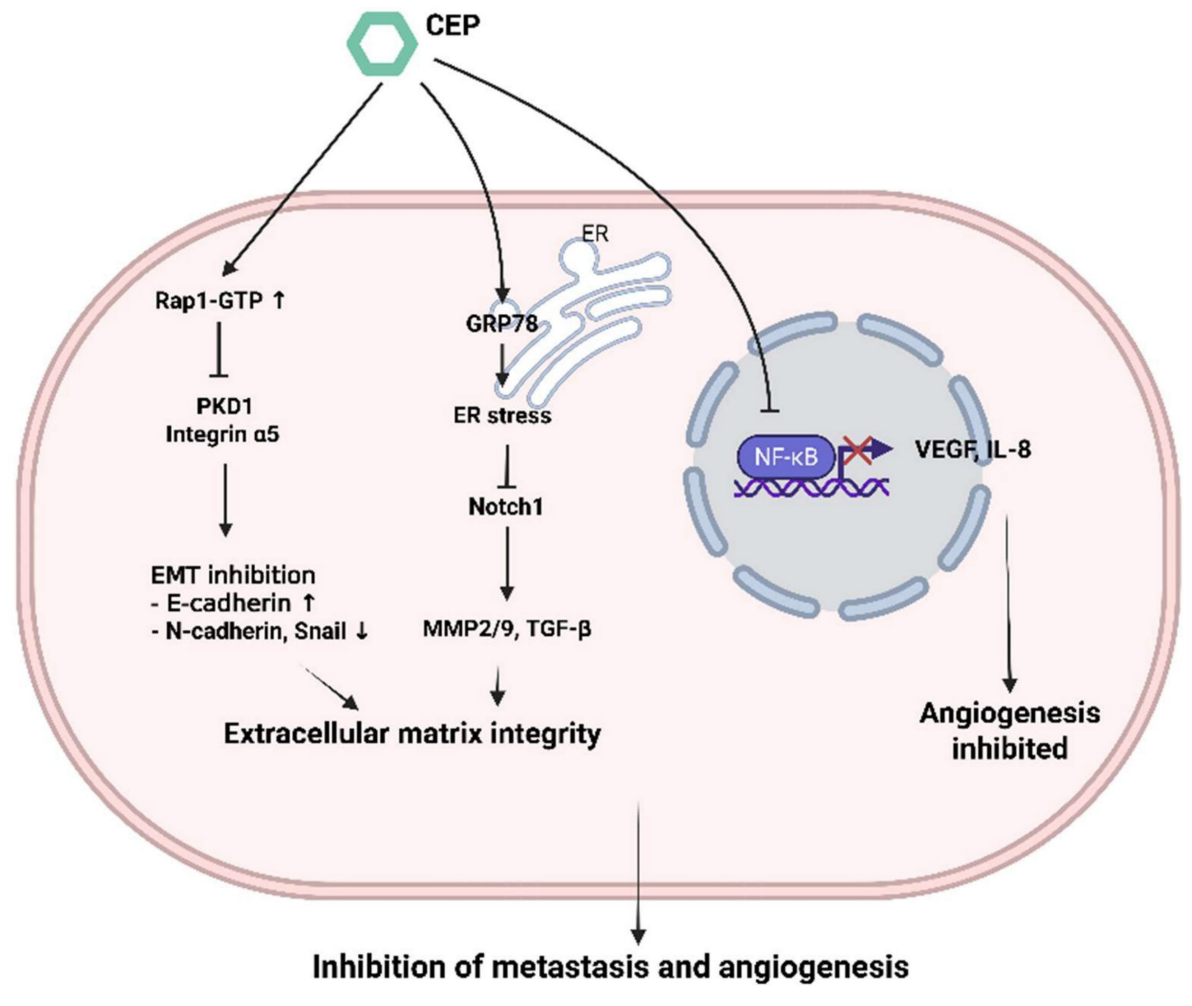
** CEP-mediated suppression of metastasis and angiogenesis through coordinated intracellular signaling.** Cepharanthine (CEP) elevates Rap1-GTP levels, which in turn inhibits PKD1 and integrin α5 signaling; this shift upregulates E-cadherin and downregulates N-cadherin and Snail to block epithelial-mesenchymal transition (EMT) and preserve extracellular matrix integrity. Simultaneously, CEP induces GRP78-dependent endoplasmic reticulum (ER) stress, leading to downregulation of Notch1 and its downstream targets MMP-2/9 and TGF-β, further stabilizing the matrix. In parallel, CEP prevents NF-κB from entering the nucleus, thereby reducing VEGF and IL-8 expression and inhibiting angiogenic sprouting. Together, these pathways converge to impede both metastatic dissemination and neovascularization. (Created with BioRender.com).

Metastasis, the spread of cancer cells from the primary tumor to distant organs, is the major cause of cancer-related mortality. It involves a complex cascade of events, including local invasion, intravasation, survival in circulation, extravasation, and colonization at a secondary site. CEP has demonstrated significant anti-metastatic properties (Figure 4).

### 4.1 Anti of Epithelial-Mesenchymal Transition

To disseminate from the primary tumor and colonize distant organs, cancer cells must first reduce intercellular adhesion and acquire enhanced migratory and invasive capabilities. This process is driven by a series of molecular and phenotypic changes, notably the downregulation of epithelial markers and upregulation of mesenchymal markers—a phenomenon known as epithelial-to-mesenchymal transition (EMT) 33, Suppression of Epithelial-Mesenchymal Transition EMT is a crucial process by which epithelial cells acquire mesenchymal characteristics, enhancing their motility and invasiveness. CEP has been shown to inhibit EMT, CEP exerts anti-bladder cancer effects by modulating the Rap1-GTP signaling pathway and altering the expression of its downstream targets, including PKD1 and ITGA5. Moreover, it regulates key proteins involved in cell migration, invasion, and epithelial-mesenchymal transition EMT 34.

### 4.2 Anti of MMPs

The role of matrix metalloproteinases MMP's in cancer metastasis has been shown to be far more complex than their functions in primary tumorigenesis. Beyond extracellular matrix degradation, MMPs can modulate a variety of molecular targets, including growth factor receptors, cytokines, chemokines, cell adhesion molecules (CAMs), apoptotic ligands, and angiogenic factors. Through these interactions, MMPs influence multiple aspects of tumor progression, such as proliferation, adhesion, migration, angiogenesis, apoptosis, and immune evasion 35, Inhibition of Matrix Metalloproteinases MMPs are enzymes that degrade the extracellular matrix ECM, facilitating cancer cell invasion, Notably, MMP2 and MMP9 are downregulated, impairing the invasive and migratory potential of cancer cells, while EMT markers such as E-cadherin, N-cadherin, and Snail are also modulated. These molecular changes collectively contribute to the inhibition of bladder cancer cell motility, invasiveness, and EMT progression, Evidence also suggests that CEP directly binds to GRP78, which contributes to the activation of ER stress and the subsequent suppression of Notch1 signaling. This suppression is evidenced by reduced cleavage of the Notch1 intracellular domain (N1ICD) and inhibition of downstream effectors, including MMP-2, MMP-9, and TGF-β, ultimately leading to the blockade of hepatocellular carcinoma lung metastasis 36.

### 4.3 Anti-Angiogenic Effects

Angiogenesis, the formation of new blood vessels from pre-existing ones, is essential for tumor growth beyond a certain size, providing oxygen and nutrients. Targeting angiogenesis is an established anticancer strategy. CEP has demonstrated anti-angiogenic properties by inhibiting key mediators of angiogenesis. In oral squamous cell carcinoma cells (B88), (HSC3), CEP suppressed angiogenesis by inhibiting the expression of Vascular Endothelial Growth Factor VEGF and Interleukin-8 IL-8. This effect was linked to the blockade of NF-κB activity 37, as NF-κB is a known transcriptional regulator of VEGF and IL-8, studies have indicated that CEP inhibited angiogenesis in endothelial cells (HUVEC) and in zebrafish in a cholesterol-dependent manner. Furthermore, CEP suppressed tumor growth *in vivo* by inhibiting angiogenesis. This could be mediated by direct effects on endothelial cells or indirectly by reducing pro-angiogenic factor secretion from tumor cells 38.

## 5. Regulation of Oxidative Stress

**Figure 5:**
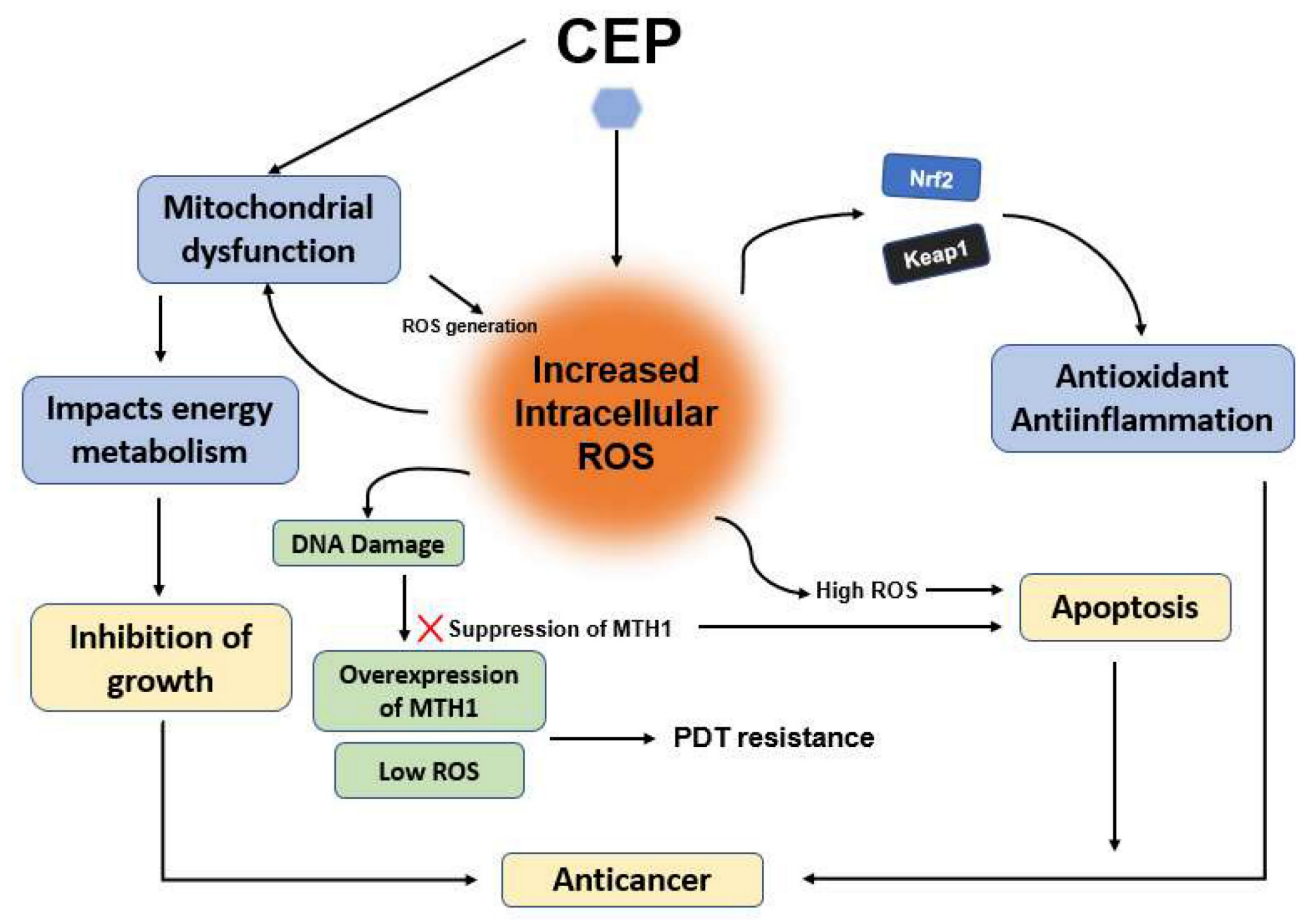
** Mechanism of CEP-induced anticancer activity through ROS modulation and defense suppression**. CEP increases intracellular ROS levels, leading to mitochondrial dysfunction and disrupted energy metabolism. This oxidative surge is further amplified by the inhibition of the Keap1-Nrf2 antioxidant pathway and the suppression of MTH1 expression, which effectively overcomes PDT resistance. Ultimately, these combined effects cause irreparable DNA damage and shift the cellular state from proliferation to apoptosis. (Created with BioRender.com).

Figure 5 shows the mechanism of CEP-induced anticancer activity through Reactive Oxygen Species (ROS) modulation and defense suppression. Reactive Oxygen Species (ROS) have a dual role in cancer. At low to moderate levels, ROS can promote cancer cell proliferation, survival, and metastasis. However, at high levels, ROS can induce oxidative stress, leading to DNA damage, protein damage, and ultimately, cell death. Many anticancer effects of CEP are associated with the generation of ROS, leading to oxidative stress-induced apoptosis. In hepatocellular carcinoma cells (Huh7, HepG2), CEP induced ROS generation, contributing to mitochondrial dysfunction and apoptosis 11, 20. Similarly, in colorectal cancer cells (HT-29, SW620), CEP was shown to generate ROS 21, in glioblastoma GBM cells (SNB-19), CEP induces ROS stress via modulation of Voltage-Dependent Anion Channel (VDAC) permeability 25. VDACs are mitochondrial channels, and their modulation can impact mitochondrial function and ROS production. CEP inhibits growth through oxidative stress and by impacting energy metabolism pathways, possibly related to its regulation of the Keap1-Nrf2 system 39, 40. The Nrf2 pathway is a primary cellular defense mechanism against oxidative stress, so its modulation by CEP could sensitize cells to ROS, in addition, the overexpression of MTH1 and insufficient ROS production are key contributors to photodynamic therapy (PDT) resistance in lung cancer cells. Treatment with CEP enhances intracellular ROS generation, resulting in DNA damage that initially triggers compensatory upregulation of MTH1. However, the combined administration of CEP and PDT induces extensive, irreparable DNA damage while concurrently suppressing MTH1 expression, ultimately leading to apoptotic cell death 41 The distinction between low/moderate ROS levels that support tumorigenesis and high ROS levels that induce apoptosis is well-established in oncology research, and CEP appears to leverage this threshold-dependent dynamic to its advantage. Furthermore, the mention of CEP's interaction with the Keap1-Nrf2 axis adds another layer of complexity. Since Nrf2 is a key transcription factor that orchestrates antioxidant responses, its inhibition by CEP could weaken the cancer cell's ability to detoxify ROS, thus amplifying the cytotoxic effect. This supports the view that CEP not only generates oxidative stress but also impairs the cellular defense against it, enhancing therapeutic efficacy, However, it's worth noting that while these findings are promising, a deeper investigation into cell-type specificity, dose dependency, and potential off-target ROS effects in normal cells is needed. Moreover, combining CEP with other treatments that modulate ROS or antioxidant defenses could be an effective strategy, but also risks inducing systemic toxicity, these findings highlight the essential role of cellular homeostasis in maintaining physiological integrity and functional stability.

## 6. Signaling Pathways

CEP's diverse anticancer effects are mediated by its ability to modulate multiple intracellular signaling pathways that are frequently dysregulated in cancer.

### 6.1 PI3K/Akt/mTOR

This pathway is a central regulator of cell growth, proliferation, survival, and metabolism, and is hyperactivated in many cancers. In nasopharyngeal carcinoma NPC cells (CNE-2, 5-8F, C666-1), CEP was shown to suppress EGFR and downstream PI3K/Akt/mTOR signaling 42, in breast cancer cells, CEP-induced autophagy, apoptosis, and G0/G1 cell cycle arrest were linked to the inhibition of the Akt/mTOR signaling pathway 13.

### 6.2 NF-κB

NF-κB is a transcription factor that plays a critical role in inflammation, immunity, cell survival, and proliferation. Its constitutive activation is common in many cancers, promoting tumorigenesis and chemoresistance. CEP suppresses the proliferation of cholangiocarcinoma cells by inhibiting the nuclear translocation of NF-κB, thereby leading to its functional inactivation 43, CEP was found to inhibit MMP-9 production in human salivary gland acinar cells, primarily through the downregulation of NF-κB signaling 44.

### 6.3 JAK2/STAT3

STAT3 is another transcription factor that is persistently activated in numerous cancers, promoting cell proliferation, survival, angiogenesis, and immune evasion. CEP suppresses proliferation and metastasis in hepatocellular carcinoma by regulating the JAK2/STAT3 pathway 11, CEP inhibits hepatocellular carcinoma progression by attenuating the JAK2/STAT3 signaling pathway, *in vivo* studies further demonstrated that CEP significantly suppressed the growth of subcutaneous HCC xenografts and reduced the expression of phosphorylated JAK2 and STAT3 in tumor tissues. These findings suggest that CEP holds potential as a therapeutic agent for HCC treatment 11.

### 6.4 Wnt/β-catenin

This pathway is crucial for embryonic development and adult tissue homeostasis. Its aberrant activation is implicated in various cancers, particularly colorectal cancer. CEP suppresses the Wnt/β-catenin signaling pathway by reducing β-catenin levels, thereby inhibiting the proliferation of colorectal cancer cells harboring APC mutations 45.

### 6.5 MAPK

MAPKs, including ERK, JNK, and p38, regulate diverse cellular processes. Their roles in cancer are complex, with ERK often promoting proliferation, while JNK and p38 can have pro-apoptotic or pro-survival functions depending on the context. A mechanistic study in hepatocellular carcinoma HuH-7 cells demonstrated that CEP induced the generation of reactive oxygen species (ROS), accompanied by the activation of key MAPK signaling pathways, including p38, JNK1/2, and ERK1/2 (p44/42), as well as the downregulation of PKB/Akt. Among these, the MAPK pathway—particularly the activation of JNK1/2—appears to play a pivotal role in mediating CEP-induced apoptosis. This suggests that MAPK signaling serves as a central hub linking oxidative stress to downstream apoptotic events, underscoring its critical involvement in the cytotoxic mechanism of CEP 20.

### 6.6 Pathway Crosstalk and Mechanistic Integration

The anticancer effects of CEP are not mediated by isolated signaling events but through intricate crosstalk among pathways. For instance, PI3K/Akt/mTOR and MAPK pathways often converge to regulate cell proliferation and survival; inhibition of Akt can sensitize cells to JNK- or p38-induced apoptosis. CEP-mediated oxidative stress has been shown to activate JNK and p38, which may further influence downstream NF-κB signaling. Additionally, NF-κB and STAT3 pathways share overlapping targets, including anti-apoptotic and inflammatory genes, and their simultaneous inhibition by CEP may synergistically suppress tumor growth and immune evasion. Crosstalk between Wnt/β-catenin and PI3K/Akt pathways has also been implicated in cancer stemness and chemoresistance, where CEP's dual inhibition potentially enhances treatment efficacy. These interactions highlight CEP's capacity to orchestrate multiple oncogenic networks, offering a comprehensive suppression of cancer cell viability and progression.

## 7. Cepharanthine Actions Across Cancer Types

The following Table 1 presents the current known effects and mechanisms of action of Cepharanthine across various cancer types; Glioblastoma (GBM), CEP (20 µM) in SNB-19 cells induces dose-dependent cytotoxicity via VDAC modulation leading to ROS stress 25. VDACs are crucial for metabolite exchange between mitochondria and cytoplasm and play a role in mitochondrial-mediated apoptosis; Oral Squamous Cell Carcinoma (OSCC), CEP (20 µg/mL) suppresses angiogenesis and growth by inhibiting VEGF and IL-8 expression through NF-κB blockade in (B88, HSC3) cells 37. This links inflammation (NF-κB, IL-8) to angiogenesis (VEGF) inhibition; Nasopharyngeal Carcinoma (NPC), CEP (15 µM) suppresses EGFR and downstream PI3K/Akt/mTOR signaling in (CNE-2, 5-8F, C666-1) cells 42. This is particularly relevant as EGFR is often overexpressed in NPC; Small Cell Lung Cancer (SCLC), CEP (10-20 µM) as a potential therapeutic agent for SCLC (H446, H1688) cells, acting through the suppression of cholesterol synthesis. This is a relatively novel mechanism for CEP's anticancer action, as cholesterol metabolism is increasingly recognized as crucial for cancer cell proliferation and membrane integrity 46; Lung Adenocarcinoma, CEP (11.2 µM) as a novel selective inhibitor of ANO1 (TMEM16A) channels in LA795 cells. ANO1 inhibition suppressed cell proliferation and migration and induced apoptosis 47. ANO1 is overexpressed in several cancers and contributes to cell proliferation, migration, and chemoresistance. In a recent study, CEP drives ferroptosis leading to anti-lung cancer efficacy through regulation of FBXL4-BNIP3 48. Gastric Cancer, CEP (10-20 µM in AGS, MKN45 cells) shows multifaceted actions, regulation of oxidative stress and energy metabolism pathways, possibly via Keap1-Nrf2 modulation 39, CEP enhances the efficacy of chemotherapeutic agents, including doxorubicin (DOX) and cisplatin (CIS). This enhancement is mediated by TRIB3's regulation of the FOXO3/FOXM1 signaling axis via the PI3K/AKT pathway, study indicate that TRIB3, in coordination with the FOXO3/FOXM1 axis, augments the therapeutic potential of CEP in the treatment of gastric cancer 49; Breast Cancer, CEP (2-20 µM) induces autophagy, apoptosis, and G0/G1 arrest via AKT/mTOR inhibition in MCF-7 and MDA-MB-231 cells) 13, CEP (2.5-10 µM) also sensitizes TNBC cells to epirubicin through cofilin oxidation-mediated mitochondrial fission and apoptosis 22; Hepatocellular Carcinoma (HCC), CEP (10-20 µM in Huh7, HepG2) inhibits proliferation and induces apoptosis through mitochondrial dysfunction and ROS generation 11, 20. In a recent study, the Cep@TCPP-MOF, developed by CEP loaded into the Tris(chlorisopropyl)Phosphate (TCPP) Metal-organic framework (MOF), is shown effect for Hepatocellular Carcinoma Sonodynamic-Chemotherapy by triggering pyroptosis through translocator protein (TSPO) Inhibition 50 ; Colorectal Cancer (CRC), CEP (5-40 µM in HT-29, SW620, HCT116, COLO-205) induces apoptosis and G0/G1 cell cycle arrest via upregulation of p21^Waf1/Cip1^, downregulation of cyclin A and Bcl-2, and ROS generation 21,CEP(25-50 µM, HeLa, C33A, CaSki) inhibits cervical cancer by inducing apoptosis and ROS-mediated oxidative stress via AMPK/p53 and Nrf2/Keap1 pathways, effectively suppressing tumor growth both in vitro and *in vivo*^40^ many evidences underscores CEP as a multifaceted anticancer agent exhibiting pleiotropic mechanisms across a broad spectrum of malignancies. A recurring theme among various cancer models is CEP's capacity to modulate oxidative stress, disrupt mitochondrial function, and regulate critical oncogenic pathways such as PI3K/Akt/mTOR, NF-κB, and EGFR.

These pathways are central to cell proliferation, survival, apoptosis, and therapeutic resistance, suggesting that CEP's pharmacological actions converge on fundamental cancer hallmarks. Furthermore, its ability to enhance the efficacy of standard chemotherapeutics—via inhibition of efflux transporters (e.g., ABCB1, ABCC10), metabolic reprogramming (e.g., cholesterol synthesis), and modulation of transcriptional regulators (e.g., TRIB3, FOXO3/FOXM1)—positions CEP as a promising adjuvant agent. Notably, CEP's activity in suppressing angiogenesis, inducing autophagy, and targeting ion channels (e.g., ANO1) reveals additional layers of therapeutic potential. These diverse mechanisms highlight CEP's translational value; however, future investigations must focus on its pharmacokinetics, bioavailability, and selectivity in vivo to fully harness its therapeutic utility and advance it toward clinical application.

## 8. Conclusion

Cepharanthine has garnered increasing attention as a natural compound with notable anticancer potential across various tumor types. Its antineoplastic activities are mediated through a range of cellular and molecular mechanisms, including the induction of apoptosis, cell cycle arrest, and inhibition of metastatic and angiogenic processes. CEP also modulates oxidative stress responses and interferes with multiple oncogenic signaling cascades, such as PI3K/Akt/mTOR, NF-κB, JAK2/STAT3, Wnt/β-catenin, and MAPK pathways. Furthermore, its capacity to counteract multidrug resistance and potentiate the action of standard chemotherapeutic agents. Accumulating experimental evidence further substantiates the potential of CEP as a multi-targeted anticancer compound. Although challenges persist, particularly regarding the optimization of its bioavailability and mechanistic precision, CEP continues to represent a compelling candidate for mechanistic studies in cancer research.

## 9. Perspectives

A major challenge in cancer chemotherapy is the development of multidrug resistance (MDR), where cancer cells become resistant to a broad range of structurally and mechanistically unrelated anticancer drugs. CEP effectively inhibits the drug transporter protein ABCC10 (also known as MRP7) on cancer cell membranes, thereby reversing drug resistance in ABCC10-expressing cells. This inhibition leads to increased intracellular accumulation of anticancer drugs by reducing their efflux from the cells 51, also impairs the activity of other drug efflux transporters, such as ABCB1 (also known as MDR1 or P-glycoprotein), which are associated with multidrug resistance in cancer cells 52, this effect may be mediated through the inhibition of the PI3K/Akt signaling pathway, resulting in decreased ABCB1 expression in cancer cells and subsequently reversing multidrug resistance (MDR) 53, CEP may directly interact with certain drug transporter proteins located on the plasma membrane. For instance, neferine—another bisbenzylisoquinoline alkaloid structurally similar to CEP—has been shown to strongly bind to and inhibit ABCB1, effectively reversing multidrug resistance (MDR). This structural similarity suggests that CEP may exert comparable biological effects 54. These findings position CEP as a compelling candidate for combination therapies aimed at overcoming MDR in various cancers. However, further studies are warranted to clarify its specificity, binding kinetics, and potential off-target effects in clinical settings. Such research will be crucial to translating these preclinical observations into effective therapeutic strategies.

CEP exerts antitumor effects via multiple pathways and targets. These mechanisms include inhibiting cell proliferation, regulating apoptosis, augmenting chemosensitivity, angiogenesis, and repressing migration and invasion, Specifically, in cervical cancer, CEP has been shown to trigger apoptosis through Bcl-2 suppression and AMPK/p53 phosphorylation while inducing cell cycle arrest, Furthermore, cepharanthine disrupted mitochondrial integrity by decreasing the mitochondrial membrane potential (∆ψm) and inducing the accumulation of reactive oxygen species (ROS), thereby driving oxidative stress 40. Collectively, these findings highlight the therapeutic potential of CEP, supporting its continued exploration in clinical contexts.

However, the clinical translation of CEP is significantly constrained by its unfavorable pharmacokinetic profile. Deng et al 16. identified a major challenge, concluding that CEP exhibits extremely poor absolute oral bioavailability (~5.65%) and a prolonged elimination half-life (*t*_1/2_ ≈ 11.02 h) in rats, indicating slow distribution and limited absorption. To facilitate bioanalytical quantification, sensitive LC-MS/MS methods were developed by Hao et al 55. and Dong et al 16. to measure CEP levels in human and rat plasma. These techniques provide essential tools for tracking systemic exposure and guiding dosage adjustments in clinical investigations.

### Further Studies

To overcome bioavailability barriers, formulation strategies using cyclodextrin microspheres have been shown to enhance CEP's water solubility, stability, and intracellular accumulation 14. Future investigations are essential to support the development of optimized preparations and ensure rational drug use.

## Funding

Financial support for this investigation was provided by Changhua Christian Hospital (Grants 112-CCH-IRP-104, 113-CCH-IRP-075 and 114-CCH-IRP-109).

## Figures and Tables

**Table 1:** Summary of the antitumor activity of cepharanthine

Cancer type	Cell line	Cep Concentration	Mechanism of Action	Reference
Glioblastoma (GBM)	SNB-19	20 µM	Induce the voltage-independent channel narrowing, which leads to the dose-dependent cytotoxicity	25
Oral Squamous Cell Carcinoma	B88, HSC3	8.2, 16.5, 33 µM	Suppress angiogenesis and growth of OSCC cells by inhibiting expression of VEGF and IL-8 involved in the blockade of NF-κB activity	37
Nasopharyngeal Carcinoma (NPC)	CNE-2, 5-8F, C666-1	15 µM	Suppresses EGFR and downstream PI3K/Akt/mTOR signaling.	42
Small cell lung cancer (SCLC)	H446, H1688	10, 20 µM	Cep as a potential therapeutic agent for SCLC, acting through the suppression of cholesterol synthesis	46
Lung adenocarcinoma	LA795	0.5, 11.2 µM	Inhibits ANO1 channels, suppressing cell proliferation and migration, and inducing apoptosis; by inhibiting FBXL4, which prevents BNIP3 ubiquitination. This stabilizes BNIP3 to activate mitophagy, subsequently driving ferroptosis to execute cancer cell death.	47, 48
Gastric cancer	AGS, MKN45	10, 20 µM	Sensitizes cells to chemotherapy by targeting TRIB3-FOXO3-FOXM1 axis to inhibit autophagy; also regulates oxidative stress and energy metabolism pathways	39, 49
Breast cancer	MCF-7, MDA-MB-231	2, 20 µM	Induces autophagy, apoptosis, and G0/G1 cell cycle arrest via inhibition of AKT/mTOR signaling pathway	13, 22
Hepatocellular carcinoma	Huh7, HepG2	10, 20 µM	Inhibits cell proliferation and induces apoptosis through mitochondrial dysfunction and ROS generation; Cep and TCPP for combined sonodynamic-chemotherapy. The synergistic interaction between Cep-mediated TSPO inhibition and TCPP-induced reactive oxygen species exacerbates mitochondrial damage, ultimately driving pyroptosis to effectively suppress hepatocellular carcinoma	11, 20, 50
Colorectal cancer	HT-29, SW620, HCT116, COLO-205	5, 40 µM	Induces apoptosis and G0/G1 cell cycle arrest via upregulation of p21^Waf1/Cip1^, downregulation of cyclin A and Bcl-2, and ROS generation	21
Cervical cancer	HeLa, C33A, CaSki	25, 50 µM	Inhibits Cervical Cancer Progression by Inducing Apoptosis and Oxidative Stress via AMPK/p53 and Nrf2/Keap1 Pathways	40
